# Population-specific gene expression in the plant pathogenic nematode *Heterodera glycines *exists prior to infection and during the onset of a resistant or susceptible reaction in the roots of the *Glycine max *genotype Peking

**DOI:** 10.1186/1471-2164-10-111

**Published:** 2009-03-16

**Authors:** Vincent P Klink, Parsa Hosseini, Margaret H MacDonald, Nadim W Alkharouf, Benjamin F Matthews

**Affiliations:** 1 Department of Biological Sciences, Harned Hall, Mississippi State University, Mississippi State, MS 39762, USA; 2 United States Department of Agriculture, Plant Sciences Institute, Beltsville, MD 20705, USA; 3 Jess and Mildred Fisher College of Science and Mathematics, Department of Computer and Information Sciences, Towson University, 7800 York Road, Towson, Maryland 21252, USA

## Abstract

**Background:**

A single *Glycine max *(soybean) genotype (Peking) reacts differently to two different populations of *Heterodera glycines *(soybean cyst nematode) within the first twelve hours of infection during resistant (R) and susceptible (S) reactions. This suggested that *H. glycines *has population-specific gene expression signatures. A microarray analysis of 7539 probe sets representing 7431 transcripts on the Affymetrix^® ^soybean GeneChip^® ^were used to identify population-specific gene expression signatures in pre-infective second stage larva (pi-L2) prior to their infection of Peking. Other analyses focused on the infective L2 at 12hours post infection (i-L2_12h_), and the infective sedentary stages at 3days post infection (i-L2_3d_) and 8days post infection (i-L2/L3_8d_).

**Results:**

Differential expression and false discovery rate (FDR) analyses comparing populations of pi-L2 (i.e., incompatible population, NL1-RHg to compatible population, TN8) identified 71 genes that were induced in NL1-RHg as compared to TN8. These genes included putative gland protein G23G12, putative esophageal gland protein Hgg-20 and arginine kinase. The comparative analysis of pi-L2 identified 44 genes that were suppressed in NL1-RHg as compared to TN8. These genes included a different Hgg-20 gene, an EXPB1 protein and a cuticular collagen. By 12 h, there were 7 induced genes and 0 suppressed genes in NL1-RHg. By 3d, there were 9 induced and 10 suppressed genes in NL1-RHg. Substantial changes in gene expression became evident subsequently. At 8d there were 13 induced genes in NL1-RHg. This included putative gland protein G20E03, ubiquitin extension protein, putative gland protein G30C02 and β-1,4 endoglucanase. However, 1668 genes were found to be suppressed in NL1-RHg. These genes included steroid alpha reductase, serine proteinase and a collagen protein.

**Conclusion:**

These analyses identify a genetic expression signature for these two populations both prior to and subsequently as they undergo an R or S reaction. The identification of genes like steroid alpha reductase and serine proteinase that are involved in feeding and nutritional uptake as being highly suppressed during the R response at 8d may indicate genes that the plant is targeting. The analyses also identified numerous putative parasitism genes that are differentially expressed. The 1668 genes that are suppressed in NL1-RHg, and hence induced in TN8 may represent genes that are important during the parasitic stages of *H. glycines *development. The potential for different arrays of putative parasitism genes to be expressed in different nematode populations may indicate how *H. glycines *evolve mechanisms to overcome resistance.

## Background

Plant parasitic nematodes are a major, but poorly understood agricultural problem, resulting in 157 billion dollars in lost revenue, annually [1-3]. The most prominent of these interactions is *G. max *infection by *H. glycines *because it accounts for an estimated $460 to $818 million in production losses annually in the U.S. [4] and approximately 15 billion dollars worldwide. Resistance loci to *H. glycines *are present in the germplasm of *G. max *and those loci have been physically mapped [5,6]. However, *G. max *resistance to *H. glycines *is limited to genotypes that are the poorest in terms of production yield. Resistance is also limited to specific nematode races (populations) within those resistant plant genotypes [7,8]. Thus, identifying genetic strategies that could confer resistance are urgently needed.

**Figure 1:**
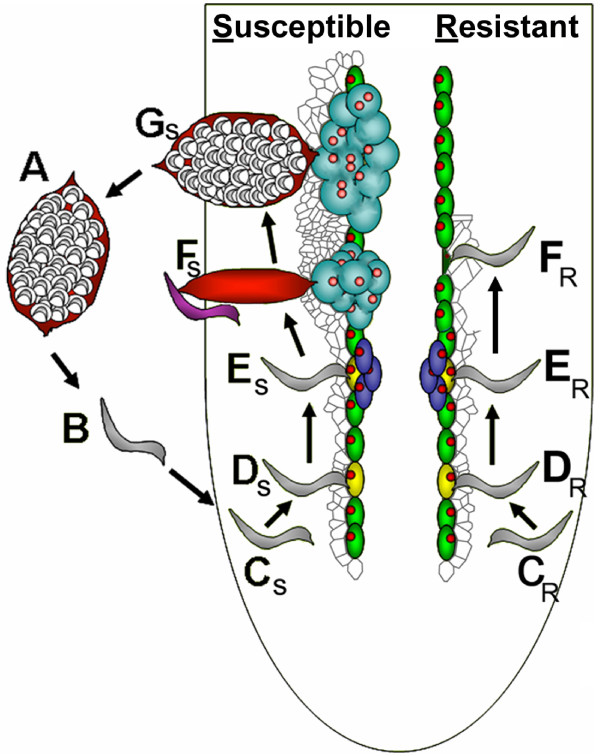
**Life cycle of *H. glycines***. **A**, cysts. **B**, pi-L2 (gray) hatch and migrate toward the root of *G. max*. **C_S_**, **C_R _**i-L2 nematodes burrow into the root and migrate toward the pericycle (green). **D_S_**, **D_R_**, i-L2 select a cell (yellow) for feeding site establishment. **E_S_**, i-L2 nematodes have molted into L3. **E_R_**, i-L2 nematodes do not increase in size. **F_S_**, The L3 undergo a subsequent molt into L4 nematodes. Meanwhile, the female continues to grow circumferentially as it feeds. The male discontinues feeding at the end of its L3 stage. Male and female L4 nematodes become adults. The vermiform male (blue) burrows outside the root and subsequently copulates with the female. **F_R_**, The syncytium collapses and the nematodes do not grow. **G**, After ~30 days, the female with eggs is clearly visible and emerging from the root. Figure adapted from Klink et al. (2008).

The *H. glycines *life cycle (Fig. 1) is approximately one month in duration [9,10]. Well-defined *H. glycines *populations that accomplish resistant (R) and susceptible (S) reactions are available for examining *G. max-H. glycines *interactions [7,8]. Those histological studies of the R and S interactions between *G. max *roots and *H. glycines *have been performed [11-18] and demonstrated the anatomical changes that occur in *G. max *roots during *H. glycines *invasion. Interestingly, nematodes burrow into the roots of *G. max *genotypes that are either resistant or susceptible. They subsequently migrate at similar rates [19,20] toward the vascular tissue, select a cell adjacent to the vascular tissue and pierce it with its stylet to initiate the development of a feeding site. This occurs at approximately 2days post inoculation (2d). Subsequently, syncytia are established during both R and S reactions. During this process, the cells adjacent to the feeding site become metabolically hyperactive [13,15]. Then, the walls of the cells adjacent to the selected cell begin to dissolve. The infected plant cell incorporates additional cells by fusion events with neighboring cells by 3d. Eventually these recruited cells merge to form a syncytium. The diverse mechanisms that accompany the R or S reaction become evident subsequently.

The S response is characterized by various cellular events that are visible at the anatomical level. These changes include hypertrophy of the nuclei and nucleoli, proliferation of cytoplasmic organelles, reduction or dissolution of the vacuole and expansion of the cell as it incorporates adjacent cells [15,16,18,21,22]. In contrast, during the R reaction of *G. max *genotype Peking to *H. glycines *NL1-RHg, the syncytium both collapses and becomes necrotic. At 4d, cell wall depositions form and there is an increase in lipid globules that occur before necrosis [18]. Concomitantly, degeneration of syncytia occurs. Bedford, a resistant genotype, has a somewhat different response. In that R reaction, nuclei first degrade. After the breakdown of the nuclei, the cytoplasm degrades [23]. Eventually, roots overcome infection at the site of infection [12,14,18,23]. These anatomical observations demonstrate that a dichotomy exists early during the development of R and S reactions and this would likely be reflected as changes in gene expression.

Genetic work in *H. glycines *has lagged behind that of model organisms. However, exciting progress has been made in generating a genetic map of *H. glycines *[24]. More recently, 454 microbead DNA sequencing has been used to generate over 400 million bases of *H. glycines *genomic sequence [25]. The work compared the genomic data from inbred avirulent (i.e., TN10) and virulent (i.e., TN20) populations of *H. glycines*. Both of those *H. glycines *biotypes, differing only by virulence, were inbred by repeated sib-mating for over 30 generations [26]. However, those populations are maintained on different plant species. TN10 was selected and maintained on *Solanum lycopersicum *(tomato) and TN20 was selected and maintained on the *H. glycines*-resistant soybean genotype Hartwig. The analysis identified 1,536 putative single nucleotide polymorphisms [25]. Thus, of 3072 PCR-validated amplicons generated between TN10 and TN20, 1108 revealed informative data for each *H. glycines *genotype [25]. *H. glycines *gene expression was not the focus of those analyses.

Microarray analysis (MA) of host-pathogen interactions can provide a broad view of gene expression during infection. Recently these sorts of genomic analyses have been adapted to the study of plant pathogenic nematodes. These studies involved determining plant gene expression in S reactions at single [27,28] or multiple time points [29-31]. However, these analyses studied only S reactions.

Experiments designed at comparing R to S reactions at multiple time points have the advantage of permitting the filtering out differential gene expression that is common between the infections caused by diverse *H. glycines *populations. This, theoretically, may allow the identification of genes expressed uniquely in the R or S reaction. Klink et al. [19,20] performed those experiments and demonstrated that while much plant gene expression was common, signature gene expression profiles existed for the R and S reactions [19,20]. This dichotomy in expression occurs early during infection and prior to the selection of feeding sites [19]. Thus, gene expression that is important to the R or S reaction is not limited to the syncytium because they have not even formed by the 12 h time point studied in that analysis [19]. Of note, a *G. max *heat shock protein 90 (HSP90) gene was found to be induced specifically during the R reaction [19]. Recent experiments using virus induced gene silencing (VIGS) of the *S. lycopersicum *HSP90 resulted in attenuation of resistance of the root knot nematode (*Meloidogyne spp*.) [32].

Signature gene expression patterns that are unique to the syncytium undergoing an R or S reaction are clearly present by 3d [20]. For example, syncytial cells have been isolated by laser capture microdissection (LCM) from roots undergoing an R reaction at 3d [20]. Those MA revealed that genes encoding lipoxygenase (LOX), HSP70 and superoxidase dismutase (SOD) were elevated almost tenfold or more [20]. In addition, genes encoding several transcription factors and DNA binding proteins were also elevated, albeit at lower levels [20].

*H. glycines *are altering gene expression of *G. max*. Thus, work on gene expression during infection in *H. glycines *may illuminate why *G. max *perceives these nematode populations differently. In the model nematode *Caenorhabditis elegans*, experiments applying MA have identified changes in gene expression during the course of its development [33,34]. Thus, MA would likely be of use to studying plant parasitic nematodes. MA of numerous previously identified esophageal gland proteins [35,36], demonstrated that it was possible to simultaneously explore plant and nematode gene expression [31]. This work set the precedent for a variety of nematode gene expression studies that go beyond the parasitism genes. The *G. max*-*H. glycines *system is powerful for the study of plant-nematode interactions. The availability of numerous well-defined *H. glycines *populations [7,8] that exhibit varying abilities to infect different *G. max *genotypes allows for the investigation of infection in ways not possible for other plant pathogenic nematodes. Prior experiments, using two different *H. glycines *populations accomplished an R (i.e., population NL1-RHg) or S reaction (i.e., population TN8) in a single *G. max *genotype (Peking) [19,20]. Thus, an S reaction can be achieved in a *G. max *genotype irrespective of the presence of resistance genes. The analyses revealed that *G. max *could perceive the two populations very differently and very early during infection, prior to feeding site selection. MA experiments that study nematode gene expression before these populations infect roots could clarify whether detectable differences are present in *H. glycines*. In this work, a comparative microarray analysis of *H. glycines *gene expression during an R and S reaction is presented.

## Methods

### Plant and nematode procurement

*H. glycines *were grown at the United States Department of Agriculture Soybean Genomics and Improvement Laboratory (SGIL), Beltsville, MD. For the analyses, incompatible (I) is defined whereby a population of nematodes successfully initiates infection, but that infection fails to proceed. This results in an R reaction. Compatible (C) is defined whereby a population of nematodes successfully initiates infection that further develops into a successful infection. This is the S reaction.

*H. glycines *were maintained in the greenhouse on the *G. max *genotype Kent using the moisture replacement system (MRS) [37]. Two populations of *H. glycines*, NL1-RHg and TN8, were used in the analyses. The *H. glycines *population, NL1-RHg, experiences an R reaction in the roots of the *G. max *genotype Peking. The *H. glycines *population TN8 experiences an S reaction in roots of the *G. max *genotype Peking.

TN8 originated by single-cyst descent on *G. max *plant introduction number 90763 (PI 90763) [26]. Originally, TN8 was maintained on the PI 90763 genotype according to standard procedures [26]. Those TN8-infected *G. max *plants were maintained in sterilized field sand medium in 1-liter containers that were suspended in a 27°C water bath. Fertilization was done with Peter's soluble 20-20-20 nutrients (The Scotts Company; Marysville, Ohio). Transfer of TN8 to a new host was performed on a 30–40 day basis. NL1-RHg has been maintained at SGIL and used extensively for analyses requiring susceptible reactions in *G. max *genotype [28,30,38] and resistant reactions in Peking [19,20,39].

Females were purified by sucrose flotation [40], and the females were crushed gently with a rubber stopper in a 7.5 cm diameter, 250 μm sieve to release the eggs. The eggs flowed through the sieve into a small plastic tray. The debris that was smaller than the eggs was removed by washing them in a 25 μm mesh sieve. The eggs were placed in a small plastic tray with one cm of water. The tray was covered with plastic wrap and placed on a rotary shaker at 25 rpm. After three days, the pre-infective second stage larvae (pi-L2) were then separated from unhatched eggs by running them through a 41 μm mesh cloth. The pi-L2s were concentrated by centrifugation in an IEC clinical centrifuge for 30 seconds at 1720 rpm and flash frozen for RNA extraction [19,41]. For studies involving infective L2 (i-L2), nematode-infected roots were collected at 12 hours post infection (h), 3 and 8 days post infection (d). The infected root tissue was flash frozen and subsequently ground in liquid N_2 _for RNA extraction [19,41]. This method has been demonstrated to be sufficient for the identification of *H. glycines *gene expression early during the early infective stages of *G. max *[31].

### Microarray analyses

Microarray gene expression analyses were conducted using the GeneChip^® ^Soybean Genome Array (Cat. # 900526; Affymetrix^®^; Santa Clara, CA) containing >7,539 *H. glycines *probe sets for 7,431 transcripts. Thus, some redundancy is present. These annotations are based on the best match from their BLASTX searches [42]. Details of the Affymetrix^® ^soybean GeneChip^® ^are available at the Affymetrix^® ^website .

The analysis presented here is different from Klink et al. [19] in that the pi-L2 stage is analyzed and more biological replicates were used. Four independent replicates were used for the NL1-RHg and TN8 pi-L2 analyses. Three independent replicates were used to analyze gene expression of the 12 h i-L2 (i-L2_12h_), 3d i-L2 (i-L2_3d_) and 8d i-L2/L3 (i-L2/L3_8d_) time points. In these analyses, the pi-L2 samples were analyzed separately from the i-L2_12h_, i-L2_3d _and i-L2/L3_8d _samples. Microarrays were hybridized and scanned at the Laboratory of Molecular Technology, SAIC-Frederick, National Cancer Institute at Frederick, Frederick, Maryland 21701, USA. Details of the scanning procedure can be found at the Affymetrix website: .

Normalization was done on the probe sets. The Affymetrix^® ^soybean GeneChip^® ^data was imported and analyzed using the MATLAB Bioinformatics Toolbox (Mathworks Inc.; Natick, MA) and ArrayAssist (Stratagene) to do RMA normalization on the probe sets before taking the log2 of expression values. Taking the log base 2 was done for scaling and compressing the data sets, as usually is done with microarray data, and not for normalizing the data sets.

Volcano plots were produced using samples having a fold change of ≥ |1.5| and also having a p-value ≤ 0.05 as compared to the control [30]. The t-test was used to calculate p-values. In addition, the differential expression analysis outcome was tested by false discovery rate (FDR), set at 10%. Significance analysis of microarrays (SAM), [43] 3.0 was used to perform FDR tests. Data supplemental to each table and figure and GO terms [44] are available .

### Heat map hierarchical clustering

The heat map was produced by mining out only the 7,539 *H. glycines *microarray probe sets on the Affymetrix^® ^soybean GeneChip^®^. Hierarchal clustering was performed using Euclidian-distance as the method of pairwise distance calculation for both the time points (columns) and the microarray probe sets (rows). The rows were clustered based on the dendrogram being two units apart; such distance being determined by the hierarchal clustering method. The resultant clusters were associated with their respective coloring. The hierarchal clustered heat map was executed in MATLAB, using both the Bioinformatics and Statistics toolboxes. All replicates for each sample type were averaged prior to clustering.

### K-means clustering

The K-means clustering [45] unsupervised learning algorithm was used for gene clustering of the three point time courses in order to find patterns in the data sets. Twelve clusters (k) were chosen for grouping the 7539 probe-set dataset. Those 12 clusters were used to identify centroids, defined as an average point specific to a cluster of points. With centroids moved into a position such that an optimum separation of objects into groups occurred, clustering was run for 1,000 repetitions using squared-Euclidian as the algorithm to measure pairwise distance between data points. Having 12 clusters fit the dataset so that all the values were affiliated with a specific cluster. Using more or fewer clusters resulted in either over-fitting or under-fitting the dataset to a respective centroid. The K-means clustering were executed in MATLAB, using both the Bioinformatics and Statistics toolboxes. All replicates for each sample type were averaged prior to clustering.

### Annotations

All annotations were obtained by performing BLASTX from the Affymetrix ID accession available at . The conserved domain search option turned on for the analyses. The best hit was used in the analyses. All annotations for the text tables and electronic supplemental tables are available at .

### Quantitative real time PCR (qRT-PCR)

Quantitative real time PCR was performed according to Klink et al. [38,46]. RNA was extracted from nematodes as previously describe [38,46]. RNA was treated with DNase I to remove genomic DNA. The cDNA was reversed transcribed from RNA using SuperScript First Strand Synthesis System for RT-PCR (Invitrogen; Grand Island, NY) with oligo d(T) as the primer according to manufacturer's instructions. Genomic DNA contamination was assessed by PCR as described previously [40]. Briefly, *Hg-unc-87 *PCR primers (forward primer: 5'GACAACACGGAGATTCCACTTCAG3'; reverse primer, 5'CTGGTCTGGTCGATGCTCTGCTC3') were used because they amplify different size fragments in the presence of genomic DNA as compared to pure cDNA. qRT-PCR reactions containing no template and reactions using RNA processed in parallel but with no Superscript reverse transcriptase also served as controls for qRT-PCR and produced no amplicon. Relative quantities of expression were determined using an Mx3000P Real-Time PCR system following manufacturer's instructions (Stratagene; La Jolla, CA). DNA accumulation was measured using SYBR Green and ROX was used as reference dye. Only one product was present in each reaction as indicated by the SYBR Green dissociation curves of amplified products and by assay of terminal reactions by gel electrophoresis in 1% TBE agarose. Template DNA was denatured for 10 minutes at 96°C, followed by PCR cycling temperatures set for denaturing for 30 seconds at 96°C, annealing for 60 seconds at 55°C and extension for 30 seconds at 72°C. The L2 stage sample was diluted over a five-log range and used in parallel RT-PCR assays. All qRT-PCR assays were conducted in triplicate. Threshold cycle (Ct) values were plotted against the dilution series. PCR efficiencies were equal between the target and endogenous control. Ct values and relative abundance were calculated using software supplied with the Mx3000P Real-Time PCR system. Standard error was used in the analyses. The qRT-PCR primers used are provided in Table 1.

**Table 1:** PCR primer pairs for qRT-PCR expression analyses.

**Afx probe set**	**Genbank ID**	**Gene**	**Primers**	**amplicon size (bp)**
HgAffx.15612.1.S1_at	CB281382	*Hg-unc-9*	F: 5'AGCCTAATGATGATCGAAACACTC3'	135
			R: 5'GAAACTGATCAGCACCGAAAATG3'	
HgAffx.18723.1.S1_at	CA940457	*Hg-unc-15*	F: 5'TTGCGGAGCTGGAAATGACC3'	105
			R: 5'GGCTGGCCTGCAACACCTT3'	
HgAffx.21154.1.S1_at	CK394306	*Hg-unc-27*	F: 5'TGGAGGAGGAGAAGTACGACATCA3'	133
			R: 5'TCATATTTGGACACTTTCTTCAGC3'	
HgAffx.17035.1.S1_at	CB279321	*Hg-unc-60B*	F: 5'AGGCGACTTTGGGGCTGGAGAG3'	121
			R: 5'ACGGCGGGGCAATTTTAGGTTC3'	
HgAffx.13291.1.S1_at	CB374691	*Hg-unc-97*	F: 5'AGAGATCGGCGGAGCACTTTAC3'	106
			R: 5'CAGCGCGGTCACCACTCTTTC3'	
HgAffx.21881.2.S1_at	CK351699	*Hg-unc-112*	F: 5'GGGCCTCCACTTGGTCACTATTAT3'	118
			R: 5'GTTCCGACATCCCTTCACTGCTC3'	
HgAffx.11541.1.S1_at	CB934909	*Hg-dys-1*	F: 5'GGGCGATGACATGCGTGACTTC3'	150
			R: 5'GCCTCTGTTTCCGCGTTCTGTGG3'	

For the PCR primers, the Genbank match for each *unc *homolog is provided. The amplicon length is provided in base pairs.

## Results

### Experimental parameters

Gene expression was examined for two populations of *H. glycines*, comparing different chronological time points of their life cycle. NL1-RHg experiences an R reaction in the roots of *G. max *genotype Peking. TN8 infection results in an S reaction in the roots of the *G. max *genotype Peking. Four time points were selected for the analysis. The first time point analyzed was a pre-infective time point. The pi-L2 is a time in *H. glycines *life cycle that occurs prior to their infection of *G. max *roots. The three remaining time points were selected from the infective stages of the life cycle. The time points that were selected for the infective stages of the life cycle are given two qualifiers, larval stage (i.e., pi-L2,) and chronological time point (i.e., 12 h, 3d or 8d). This was done because some stages of the nematode life cycle last for numerous days and gene expression (i.e., *uncoordinated *[*unc*] gene expression) changes during the course of a given stage [46]. Therefore, the time points (T) in the analyses are pi-L2 (T1) (Fig. 1, stage B), i-L2 at 12 h (T2) (Fig. 1, stage CR and CS), i-L2 at 3d (T3) (Fig. 1, stage ER and ES) and i-L2/L3 at 8d (T4) (Fig. 1, stage FR and FS). It is assumed that the T4 samples are i-L2/L3 according to (Jenkins and Taylor 1967 [9]). For the presentation of gene expression, fold change is used even though FDR was performed as an alternative way to identify differentially expressed genes.

### Gene expression-time point analyses that directly compare R to S reactions

**Figure 2:**
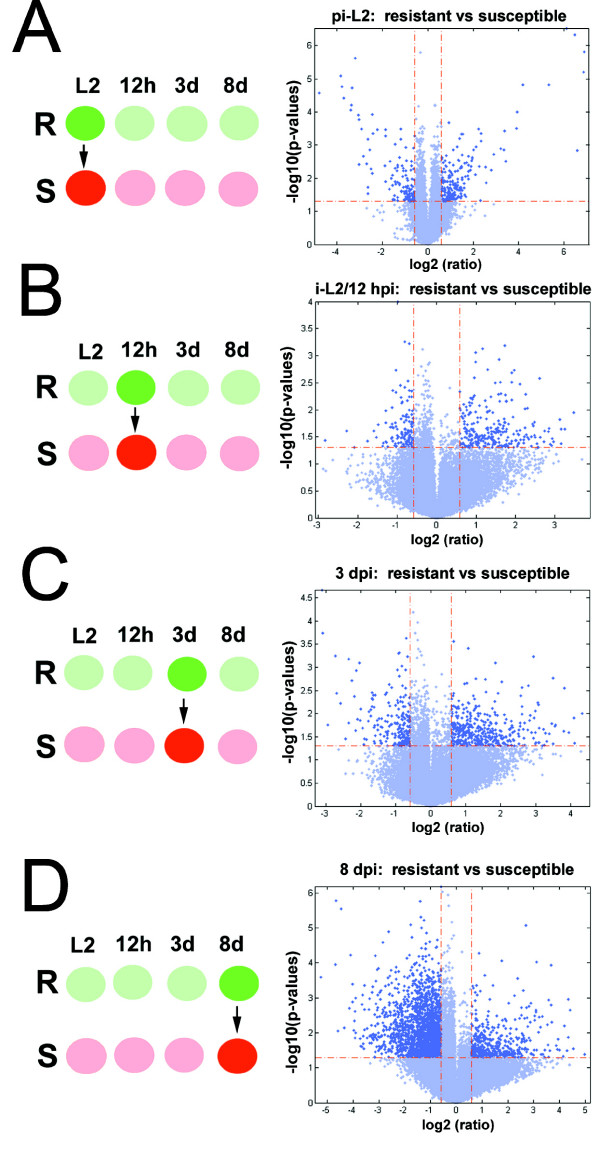
**Volcano plots comparing differential gene expression between NH1-RHg (R) and TN8 (S) sample types**. A cutoff of ≥ |1.5| fold cutoff and P ≤ 0.05 was used for the analyses. Induced genes (dark blue, upper right quadrant) are genes induced during the resistant reaction. Suppressed genes (dark blue, upper left quadrant) are genes suppressed during the resistant reaction. The differentially expressed genes are presented in context for NH1-RHg. **A**, L2 (pi-L2); **B**, 12 h (i-L2_12h_); **C**, 3d (i-L2_3d_); **D**, 8d (i-L2/L3_8d_).

In the first set of experiments, *H. glycines *gene expression was examined in the R reaction. This was done by directly comparing R expression to S expression. The analysis directly compared R to S at T1, T2, T3 and T4 (Fig. 2). The S reaction was used as the baseline at each time point by which gene expression in the R reaction was determined. In all analyses, a ≥ |1.5| fold expression limit with a p-value of ≤ 0.05 was used. Volcano plots (Fig. 2) depict differential gene expression of all probe sets on the Affymetrix^® ^soybean GeneChip^® ^at T1 (Fig. 2A), T2 (Fig. 2B), T3 (Fig. 2C) and T4 (Fig 2D). The analysis presented here measures genes induced in the R reaction. Conversely, due to the nature of the volcano plot analysis, those same induced genes in R would be suppressed by the same measure in the S reaction.

The first analysis presents *H. glycines *gene expression in R at T1. This analysis identified 71 genes (~0.94% of the probe sets) were induced in NL1-RHg as compared to TN8 (Table 2, Supplementary Table One). A large percentage (71.83%) had no match to known genes in Genbank. Of those, 19 (26.76%) were induced by five-fold or greater. The analysis also identified 44 genes (~0.58% of the probe sets) that were suppressed in NL1-RHg as compared to TN8 (Table 2, Supplementary Table One). A large percentage (63.64%) had no match to known genes in Genbank. Of those, 21 (47.73%) were suppressed five-fold or greater (p-value ≤ 0.05 FDR set at 10%). Of note, but not presented in the analysis was the identification of two genes annotated to be bacterial in homology. This included a cell wall hydrolase (CK348492) from the bacterium *Burkholderia multivorans *ATCC 17616 that was suppressed -6.23 fold (p-value of 0.0294, FDR of 11.61042). (Table 2, Supplementary Table One).

**Table 2:** Differentially expressed genes in pi-L2.

**R-pi-L2-INDUCED**								
**Afx ID**	**Array ID**	**Best Hit ID**	**E-value**	**Best Hit Organism**	**Best Hit Description**	**FC**	**P-Value**	**q-Value(%)**
HgAffx.22005.3.S1_at	AF500033.1	AAP30772	1.00E-56	Heterodera glycines	putative gland protein G23G12	117	0.00000611	0
HgAffx.22005.3.S1_x_at	AF500033.1	AAP30772	1.00E-56	Heterodera glycines	putative gland protein G23G12	69.3	0.000000293	0
HgAffx.12397.1.S1_at	CB375585	AAL78214	8.00E-12	Heterodera glycines	putative esophageal gland cell protein Hgg-20	9.21	0.00125	0
HgAffx.10464.1.S1_at	CB378037	NP_001023126	4.00E-12	Caenorhabditis elegans	Temporarily Assigned Gene name (tag-287)	7.52	0.00188	0
HgAffx.6167.1.S1_at	CB826044	AAL78214	3.00E-14	Heterodera glycines	putative esophageal gland cell protein Hgg-20	5.04	0.000766	0
HgAffx.13471.7.S1_at	BF013637	AAO49799	7.00E-36	Heterodera glycines	arginine kinase	4.71	0.000329	0
HgAffx.15037.1.S1_at	CB375286	XP_001900724	4.00E-15	Brugia malayi	BESS motif family protein	4.26	0.00792	1.562478529
HgAffx.23436.1.S1_at	BF014413	AAP30770	2.00E-11	Heterodera glycines	putative gland protein G17G01	3.86	0.00254	0
HgAffx.3818.1.A1_at	CK348872	XP_001902079	2.00E-16	Brugia malayi	Fucosyl transferase family protein	3.63	0.00575	1.562478529
HgAffx.22919.1.S1_at	CB375623	XP_001670157	2.00E-15	Caenorhabditis briggsae AF16	Hypothetical protein CBG06224	3.48	0.0104	1.562478529
HgAffx.4275.2.S1_at	AF500024.1	AAP30763	4.00E-170	Heterodera glycines	putative gland protein G8H07	3.05	0.00348	0
HgAffx.9246.1.S1_at	CB379256	XP_001899138	1.00E-72	Brugia malayi	3'-5' exonuclease family protein	3.03	0.0199	9.06375819
HgAffx.11497.1.S1_at	CB934953	XP_001900969	3.00E-24	Brugia malayi	DNA methyltransferase 1 associated protein 1	2.73	0.0189	9.06375819
HgAffx.15350.1.S1_at	CB281644	XP_001196614	6.00E-53	Strongylocentrotus purpuratus	Serine/threonine-protein kinase Eg2-like	2.51	0.0028	1.562478529
HgAffx.23782.1.S1_at	BF014067	XP_001899293	5.00E-13	Brugia malayi	hypothetical protein Bm1_39195	2.5	0.00606	9.06375819
HgAffx.2353.1.S1_at	CK350675	YP_001958273	2.00E-13	Candidatus Amoebophilus asiaticus 5a2	hypothetical protein Aasi_1217	2.16	0.0014	1.562478529
HgAffx.1966.1.S1_at	CK351813	AAO85518	1.00E-59	Oesophagostomum dentatum	putative serine/threonine phosphatase	1.88	0.000209	0
HgAffx.12364.1.S1_at	AF344864.1	AAL78213	5.00E-139	Heterodera glycines	probable protein kinase Hgg-20	1.77	0.00444	9.06375819
HgAffx.6833.1.S1_at	CB825378	XP_001901483	2.00E-36	Brugia malayi	hypothetical protein Bm1_50075	1.69	0.00296	9.06375819
**R-pi-L2-SUPPRESSED**								
**Afx ID**	**Array ID**	**Best Hit ID**	**E-value**	**Best Hit Organism**	**Best Hit Description**	**FC**	**P-Value**	**q-value(%)**
HgAffx.22522.2.A1_s_at	CK351795	AAL78214	6.00E-30	Heterodera glycines	putative esophageal gland cell protein Hgg-20	-14.5	0.000008	0
HgAffx.22522.2.A1_x_at	CK351795	AAL78214	6.00E-30	Heterodera glycines	putative esophageal gland cell protein Hgg-20	-13.9	0.0000174	0
HgAffx.22522.2.A1_at	CK351795	AAL78214	6.00E-30	Heterodera glycines	putative esophageal gland cell protein Hgg-20	-13.1	0.0000382	0
HgAffx.8428.1.S1_at	CB825226	XP_001896306	7.00E-28	Brugia malayi	PX domain containing protein	-8.28	0.000124	0
HgAffx.2485.1.S1_at	CK350205	XP_001677586	4.00E-23	Caenorhabditis briggsae AF16	Hypothetical protein CBG05236	-7.66	0.000329	0
HgAffx.22522.2.S1_at	CK351795	AAL78214	6.00E-30	Heterodera glycines	putative esophageal gland cell protein Hgg-20	-6.48	0.00364	0
HgAffx.22522.2.S1_x_at	CK351795	AAL78214	6.00E-30	Heterodera glycines	putative esophageal gland cell protein Hgg-20	-5.64	0.00533	0
HgAffx.20464.1.S1_at	BI749289	NP_505424	7.00E-27	Caenorhabditis elegans	E02C12.4	-4.68	0.000339	0
HgAffx.13898.1.S1_at	CB378644	NP_495784	1.00E-32	Caenorhabditis elegans	ZYGote defective (zyg-9)	-3.67	0.00052	0
HgAffx.10090.1.S1_at	CB378411	CAB88203	2.00E-28	Globodera pallida	putative cuticular collagen	-3.65	0.0128	5.390550924
HgAffx.12914.1.S1_at	CB281819	XP_001665811	1.00E-54	Caenorhabditis briggsae AF16	hypothetical protein CBG18196	-3.65	0.000339	0
HgAffx.15942.1.S1_at	CB280414	XP_001676377	5.00E-11	Caenorhabditis briggsae AF16	Hypothetical protein CBG14167	-2.74	0.0049	1.268364923
HgAffx.21565.2.S1_at	BI748188	XP_001678481	2.00E-14	Caenorhabditis briggsae AF16	Hypothetical protein CBG13418	-2.52	0.00454	1.268364923
HgAffx.15681.1.S1_at	CB281313	XP_001678446	1.00E-21	Caenorhabditis briggsae AF16	Hypothetical protein CBG13461	-2.19	0.00417	4.791600821
HgAffx.23342.1.S1_at	BF014507	CAC83611	7.00E-61	Globodera rostochiensis	EXPB1 protein	-2.16	0.00882	9.06375819

Probe set lists that are induced in pi-L2 NL1-RHg that ultimately undergo a resistant reaction. FC- NL1-RHg, fold change- NL1-RHg; PV- NL1-RHg, p-value-NL1-RHg. A > |1.5| fold cutoff and P ≤ 0.05 was used. FDR (set at 10%) parameters are: q-Value (%).

The second analysis presents *H. glycines *gene expression in R at T2. This analysis identified 7 genes (~0.09% of the probe sets) that were induced in NL1-RHg as compared to TN8 (Table 3, Supplementary Table Two). A small percentage (14.29%) had no match to known genes in Genbank. Of those, none were induced by five-fold or greater. The most highly induced gene was actin (AAG47837) isolated from *H. glycines*. The analysis also identified 0 genes that were suppressed in NL1-RHg as compared to TN8 (p-value ≤ 0.05 FDR set at 10%) (Table 3, Supplementary Table Two). (Table 3, Supplementary Table Two).

**Table 3:** Differentially expressed genes in i-L2 NL1-RHg at 12 h.

**R-12 hour-INDUCED**								
**Afx ID**	**Array ID**	**Best Hit ID**	**E-value**	**Best Hit Organism**	**Best Hit Description**	**FC**	**P-Value**	**q-value(%)**
AFFX-r2-Hg-actin-3_x_at	AF318603.2	AAG47837	0	Heterodera glycines	actin 1	4.78	0.00513	0
HgAffx.15051.1.S1_at	CB279435	ABN64198	2.00E-22	Meloidogyne incognita	glutathione S-transferase-1	3.38	0.00692	0
AFFX-r2-Hg-actin-3_at	AF318603.2	AAG47837	0	Heterodera glycines	actin 1	3	0.00117	0
HgAffx.18740.1.S1_at	AF318603.2	AAG47837	0	Heterodera glycines	actin 1	2.8	0.00318	0
HgAffx.18740.1.S1_x_at	AF318603.2	AAG47837	0	Heterodera glycines	actin 1	2.59	0.00582	0
HgAffx.22081.1.S1_at	CK349167	NP_496721	4.00E-30	Caenorhabditis elegans	Carnitine Palmitoyl Transferase family member (cpt-1)	2.46	0.0018	0
**R-12 hour-SUPPRESSED**								
**Afx ID**	**Array ID**	**Best Hit ID**	**E-value**	**Best Hit Organism**	**Best Hit Description**	**FC**	**P-Value**	**q-value(%)**

Probe set lists that are induced i-L2 NL1-RHg at 12 h that ultimately undergo a resistant reaction. FC-NL1-RHg; fold change- NL1-RHg; PV- NL1-RHg, p-value-NL1-RHg. A > |1.5| fold cutoff and P ≤ 0.05 was used. FDR (set at 10%) parameters are: Score (d), q-value (%).

The third analysis presents *H. glycines *gene expression in R at T3. This analysis identified 9 genes (~0.12% of the probe sets) that were induced in NL1-RHg as compared to TN8 (Table 4, Supplementary Table Three). A large percentage (77.78%) had no match to known genes in Genbank. Of those, one (11.11%) was induced by five-fold or greater. The analysis also identified 7 genes (~0.09% of the probe sets) that were suppressed in NL1-RHg as compared to TN8 (Table 4, Supplementary Table Three). Approximately 14.28% had no match to known genes in Genbank. Of those, one (CD748082), a predicted protein from *Nematostella vectensis *(starlet sea anemone) was suppressed by five-fold or greater (p-value ≤ 0.05 FDR set at 10%) (p-value ≤ 0.05) (Table 4, Supplementary Table Three).

**Table 4:** Differentially expressed genes in i-L2 NL1-RHg at 3d.

**R-3 DAY-INDUCED**								
**Afx ID**	**Array ID**	**Best Hit ID**	**E-value**	**Best Hit Organism**	**Best Hit Description**	**FC**	**P-Value**	**q-value(%)**
HgAffx.20336.1.S1_at	AF044210.1	AAC33848	2.00E-172	Heterodera glycines	beta-1,4-endoglucanase-3 precursor	3.41	0.0412	9.206618641
AFFX-r2-Hg-actin-3_at	AF318603.2	AAG47837	0	Heterodera glycines	actin 1	2.18	0.0103	9.206618641
								
**R-3 DAY-SUPPRESSED**								
**Afx ID**	**Array ID**	**Best Hit ID**	**E-value**	**Best Hit Organism**	**Best Hit Description**	**FC**	**P-value**	**q-value(%)**
HgAffx.17178.1.S1_at	CD748082	XP_001641607	2.00E-12	Nematostella vectensis	predicted protein	-8.5	0.000186	8.613475143
HgAffx.16643.1.S1_at	CK349745	XP_001895036	6.00E-20	Brugia malayi	T-complex protein 1, delta subunit	-4.24	0.013	9.206618641
HgAffx.22600.1.S1_at	AF469055.1	AAN32884	0	Heterodera glycines	cellulase ENG-5	-1.95	0.0109	4.774861221
HgAffx.20813.1.S1_at	AF502393.1	AAP30836	3.00E-83	Heterodera glycines	putative gland protein G30C02; Hg-G30C02	-1.58	0.027	0
HgAffx.2725.1.S1_at	AF490251.1	AAO85459	1.00E-103	Heterodera glycines	putative gland protein G20E03	-1.53	0.0121	4.774861221
HgAffx.9968.2.S1_at	CB374502	XP_001664903	8.00E-54	Caenorhabditis briggsae AF16	hypothetical protein CBG24609	-1.52	0.0392	9.206618641

Probe set lists that are induced in NL1-RHg at 3d that ultimately undergo a resistant reaction. FC-NL1-RHg, fold change-NL1-RHg; PV-NL1-RHg, p-value-NL1-RHg. A> |1.5| fold cutoff and P ≤ 0.05 was used. FDR (set at 10%) parameters are: Score (d), q-value (%).

The fourth analysis presents *H. glycines *gene expression in R at T4. This analysis identified 13 genes (~0.17% of the probe sets) that were induced in NL1-RHg as compared to TN8 (Table 5, Supplementary Table Four). A large percentage (46.15%) had no match to known genes in Genbank. Of those, 11 (84.62%) were induced by five-fold or greater. The most highly induced gene was a putative gland protein G20E03 (AF490251.1) from *H. glycines*. The analysis also identified a substantial number of genes (N = 1668 [~22.24% of the probe sets]) that were suppressed in NL1-RHg as compared to TN8 (Table 5, Supplementary Table Four). A large percentage (33.5%) had no match to known genes in Genbank. Of those, 176 (10.55%) were suppressed five-fold or greater (p-value ≤ 0.05) (Table 5, Supplementary Table Four). While several highly suppressed genes were identified, the two most highly suppressed genes of known function were a steroid alpha reductase (CB825108) and a serine protease (Y13906.1) (p-value ≤ 0.05 FDR set at 10%).

**Table 5:** Differentially expressed genes in i-L2 NL1-RHg at 8d.

**R-8 DAY-INDUCED**								
**Afx ID**	**Array ID**	**Best Hit ID**	**E-value**	**Best Hit Organism**	**Best Hit Description**	**FC**	**P-value**	**q-value(%)**
HgAffx.2725.1.S1_at	AF490251.1	AAO85459	1.00E-103	Heterodera glycines	putative gland protein G20E03	12.7	0.000118	0
HgAffx.22770.1.S1_at	AF469060.1	AAN32889	2.00E-45	Heterodera glycines	ubiquitin extension protein	9.22	0.00353	1.523165321
HgAffx.20740.1.S1_at	CD748921	NP_498657	1.00E-26	Caenorhabditis elegans	R13A5.6	9.18	0.00964	6.920221232
HgAffx.20813.1.S1_at	AF502393.1	AAP30836	3.00E-83	Heterodera glycines	putative gland protein G30C02; Hg-G30C02	8.73	0.0000945	0
HgAffx.20336.1.S1_at	AF044210.1	AAC33848	2.00E-172	Heterodera glycines	beta-1,4-endoglucanase-3 precursor	8.55	0.00254	1.068579553
HgAffx.20336.2.S1_s_at	AY043224.1	AAK85303	0	Heterodera glycines	beta-1,4-endoglucanase-4	6.66	0.00374	1.912614015
HgAffx.22005.1.S1_s_at	AF502392.1	AAP30835	1.00E-136	Heterodera glycines	putative gland protein G33E05; Hg-G33E05	2.99	0.0032	1.523165321
								
**R-8 DAY-SUPPRESSED**								
**Afx ID**	**Array ID**	**Best Hit ID**	**E-value**	**Best Hit Organism**	**Best Hit Description**	**FC**	**P-value**	**q-value(%)**
HgAffx.17401.1.S1_at	CB825108	NP_495430	2.00E-84	Caenorhabditis elegans	steroid Alpha ReducTase family member (art-1)	-17	0.0000588	0
HgAffx.7076.1.S1_at	Y13906.1	CAA74204	2.00E-158	Heterodera glycines	serine proteinase	-16.4	0.000412	0
HgAffx.1292.1.S1_at	CB378350	NP_783594	1.00E-21	Mus musculus	histone cluster 1, H2ba	-15.5	0.00451	0.280100042
HgAffx.14690.2.S1_at	CB826267	XP_001668392	6.00E-34	Caenorhabditis briggsae AF16	Hypothetical protein CBG12388	-15.2	0.00248	0.177088891
HgAffx.19766.1.S1_at	CB375606	AAG21338	4.00E-12	Heterodera glycines	hypothetical esophageal gland cell secretory protein 9	-12.7	0.00155	0.177088891
HgAffx.22425.1.S1_at	BI396655	NP_501123	1.00E-19	Caenorhabditis elegans	COLlagen family member (col-113)	-12.5	0.000818	0.177088891
HgAffx.2920.1.S1_at	CK349770	AAL78218	5.00E-48	Heterodera glycines	histone Hgg-28	-12.1	0.0124	0.60765796
HgAffx.17467.1.S1_at	CB299232	NP_509186	4.00E-15	Caenorhabditis elegans	Carbonic AnHydrase family member (cah-5)	-11.9	0.000438	0
HgAffx.3069.1.S1_at	CK350667	XP_001175793	2.00E-18	Strongylocentrotus purpuratus	PREDICTED: similar to histone H2A	-11.7	0.00802	0.48372909
HgAffx.18866.1.S1_at	CA940314	CAB88204	5.00E-20	Globodera pallida	putative cuticular collagen	-11.7	0.00213	0.177088891
HgAffx.17449.1.S1_at	CK348660	XP_001541426	5.00E-16	Ajellomyces capsulatus NAm1	histone H2A	-11.1	0.0143	0.817748094
HgAffx.2088.1.S1_at	CK351923	XP_001668016	2.00E-25	Caenorhabditis briggsae AF16	Hypothetical protein CBG03859	-11.1	0.00765	0.41363165
HgAffx.2863.1.S1_at	CK349827	AAR85527	6.00E-46	Meloidogyne incognita	14-3-3b protein	-10.3	0.0195	1.068579553
HgAffx.11150.1.S1_at	CB378957	NP_508280	3.00E-96	Caenorhabditis elegans	D1005.1	-10.3	0.00107	0.177088891
HgAffx.7347.1.S1_at	CK394435	XP_001672273	4.00E-19	Caenorhabditis briggsae AF16	Hypothetical protein CBG11380	-10.2	0.0123	0.60765796
HgAffx.10212.1.S1_at	CB378289	XP_001678368	1.00E-12	Caenorhabditis briggsae AF16	Hypothetical protein CBG21909	-9.72	0.0062	0.280100042
HgAffx.11037.1.S1_at	CB935417	XP_001670890	0	Caenorhabditis briggsae AF16	Hypothetical protein CBG19828	-9.63	0.000703	0.177088891
HgAffx.19641.1.S1_at	CD748996	XP_001895768	6.00E-22	Brugia malayi	Probable 3-hydroxyacyl-CoA dehydrogenase B0272.3	-9.62	0.0112	0.60765796
HgAffx.1801.1.S1_at	CK350930	XP_001429543	1.00E-36	Paramecium tetraurelia strain d4-2	hypothetical protein GSPATT00032377001	-9.21	0.0156	0.913385871
HgAffx.9435.1.S1_at	CB379066	XP_001673815	3.00E-59	Caenorhabditis briggsae AF16	Hypothetical protein CBG23138	-9.16	0.0127	0.60765796

Probe set lists that are induced in NL1-RHg at 8d that ultimately undergo a resistant reaction. FC-NL1-RHg, fold change-NL1-RHg; PV-NL1-RHg, p-value-NL1-RHg. A> |1.5| fold cutoff and P ≤ 0.05 was used. FDR (set at 10%) parameters are: Score (d), q-value (%).

### qRT-PCR-mediated validation

Validation was performed. This was done by a qRT-PCR analysis. *H. glycines *gene expression from the microarray analysis was determined to correlate with that obtained from previous qRT-PCR analyses [46] (Table 6).

**Table 6:** RT-PCR validation

**Afx probe set**	**Genbank ID**	**Gene**	**MA-L2**	**qRT-PCR-L2**
HgAffx.13291.1.S1_at	CB374691	*Hg-unc-97*	1.6239316	1.284
HgAffx.18723.1.S1_at	CA940457	*Hg-unc-15*	1.2863248	1.274
HgAffx.21154.1.S1_at	CK394306	*Hg-unc-27*	1.2735043	1.253
HgAffx.21881.2.S1_at	CK351699	*Hg-unc-112*	1.2521368	1.139
HgAffx.15612.1.S1_at	CB281382	*Hg-unc-9*	1.1410256	1.435
HgAffx.11541.1.S1_at	CB934909	*Hg-dys-1*	1.4358974	1.442
HgAffx.17035.1.S1_at	CB279321	*Hg-unc-60B*	1.4444444	1.419

Column headings: Afx probe set, GeneChip^® ^array identifier; Genbank ID, Genbank accession number; L2-qRT-PCR, L2 fold expression from qRT-PCR; L2-MA, L2 fold expression on microarray.

### Gene expression: analyses within R or S reaction types that are between time points

**Figure 3:**
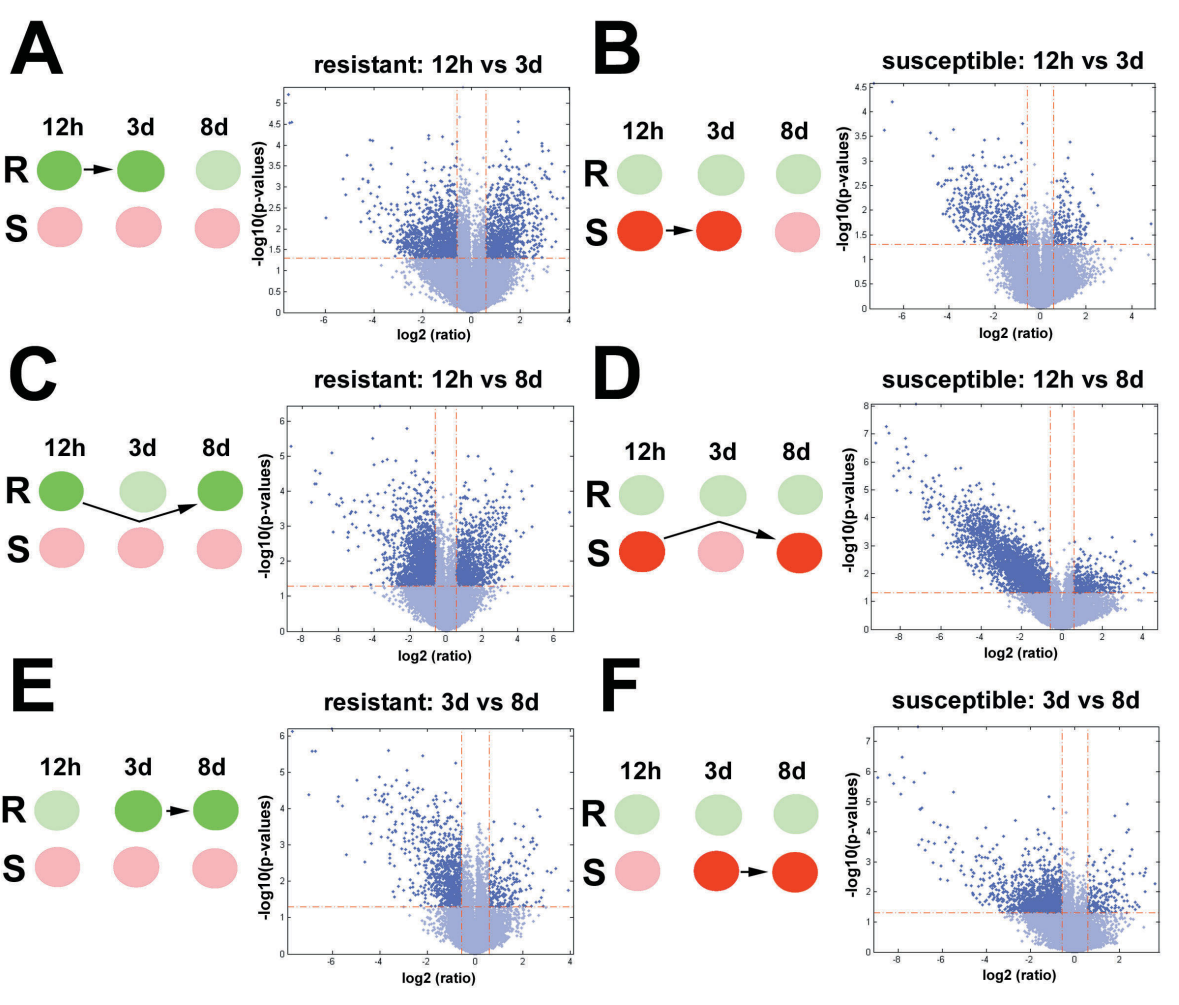
**Volcano plots comparing differential gene expression between resistant or susceptible sample types at different stages of infection**. A cutoff fold change of |1.5| and p-value of </= 0.05 was used for the analyses. Induced genes (dark blue, upper right quadrant) are genes induced during the resistant reaction. Suppressed genes (dark blue, upper left quadrant) are genes suppressed during the resistant reaction. **A-C**, resistant comparisons. **D-F**, susceptible comparisons. **A**, NL1-RHg_12h _vs NL1-RHg_3d_; **B**, NL1-RHg_12h _vs NL1-RHg_8d_; **C**, NL1-RHg_3d _vs NL1-RHg_8d_; **D**, TN8_12h _vs TN8_3d_; **E**, TN8_12h _vs TN8_8d_; **F**, TN8_3d _vs TN8_8d_.

Gene expression was analyzed across time points within a reaction type (i.e., R or S) (Fig. 3). The analyses compared the R reactions between 12 h and 3d (Fig. 3A; Supplementary Table Five) and S reactions between 12 h and 3d (Fig. 3B; Supplementary Table Six). The analyses then compared R reactions between 12 h and 8d (Fig. 3C; Supplementary Table Seven) and S reactions between 12 h and 8d (Fig. 3D; Supplementary Table Eight). The analyses concluded by comparing R reactions between 3d and 8d (Fig. 3E; Supplementary Table Nine) and S reactions between 3d and 8d (Fig. 3F; Supplementary Table Ten). The differential expression outcomes were further substantiated by FDR.

Comparing the 12 h R reaction (baseline) to the 3d R reaction revealed differentially expressed genes in the 3d R reaction (Fig. 3A; Supplementary Table Five). Only one induced gene of unknown function (BF014388) (fold change [FC]: 1.58) was identified. The five most highly suppressed genes found were the gland-specific protein G4G12 (AF473827.1), (FC: -182); a hypothetical gene (CK348411), (FC: -167); a cathepsin S-like cysteine proteinase (Y09499.1), (FC: -34.6); a gland-specific protein G4E02 (AF473826.1), (FC: -21.8) and an unknown (CB280439), (FC: -19).

Comparing the 12 h S reaction (baseline) to the 3d S reaction revealed differentially expressed genes in the 3d S reaction (Fig. 3B; Supplementary Table Six). Induced genes were a hypothetical protein (CB826489), (FC: 28.5); a GTP-binding nuclear protein, RAN/TC4 (CB378243), (FC: 7.15); a snRNP protein (CB279314), (FC: 5.68); a cathepsin B-like proteinase (CB278894), (FC: 5.27) and a cathepsin S-like cysteine proteinase (Y09499.1), (FC: 4.81). Suppressed genes were an *H. glycines *expressed sequence tag (EST) (CK351599), (FC: -115) lacking homology to any gene; a homolog to the F08F8.7 gene (BF014655), (FC: -25.9); an elongation factor 1-alpha (EF-1-alpha), (BI749209) (FC: -22); a 60S ribosomal protein L39 (CA939898), (FC: -20.1) and a putative gland protein 29D09 (AF500016.1), (FC: -17.9).

Comparing the 12 h R reaction (baseline) to the 8d R reaction revealed differentially expressed genes in the 8d R reaction (Fig. 3C; Supplementary Table Seven). Several induced genes of unknown function were identified. Other induced genes found were a beta-1,4-endoglucanase-4 (AY043224.1), (FC: 4.92) and a cellulase (ENG-5), (AF469055.1), (FC: 4.72). Highly suppressed genes found were a putative cuticular collagen (CAB88203), (FC: -317); a C-type lectin domain protein (AF498244.1), (FC: -155); a hypothetical protein (CK348411), (FC: -151); the hypothetical protein Hgg-18 (CB935297), (FC: -89.7); the hypothetical esophageal gland cell secretory protein 4 (AF273731.2), (FC: -68.7) and the Y52B11A.8 gene (CB281657), (FC: -61.8).

Comparing the 12 h S reaction (baseline) to the 8d S reaction revealed differentially expressed genes in the 8d S reaction (Fig. 3D; Supplementary Table Eight). Induced genes found included a beta 1,4-endoglucanase-4 (AY043224.1), (FC: 8.12); a beta-1,4-endoglucanase-3 precursor (AF044210.1), (FC: 6.53); the expansin, EXPB1, (BF014507), (FC: 3.16) and a beta-1,4-endoglucanase-4 (AY043224.1), (FC: 2.74). Highly suppressed genes were a putative cuticular collagen (CAB88203), (FC: -603); a C-type lectin domain protein (AF498244.1), (FC: -414); a hypothetical protein Hgg-18 (CB935297), (FC: -289); a hypothetical esophageal gland cell secretory protein 4 (AF273731.2), (FC: -282); a hypothetical protein (CK348411), (FC: -232) and the Y52B11A.8 gene (CB281657), (FC: -216).

Comparing the 3d R reaction (baseline) to the 8d R reaction revealed differentially expressed genes in the 8d R reaction (Fig. 3E; Supplementary Table Nine). Induced genes found included the gland-specific protein G4G05 (AF473830.1), (FC: 2.84) and the expansin, EXPB1, (BF014507), (FC: 2.84). Suppressed genes were a C-type lectin domain protein (AF498244.1), (FC: -115); the hypothetical esophageal gland cell secretory protein 4 (AF273731.2), (FC: -55.2); the hypothetical Y52B11A.8 gene (CB281657), (FC: -53.6); a putative cuticular collagen (CB378944), (FC: -47.9) and the hypothetical protein CBG21909 (CB378289), (FC: -12.1).

Comparing the 3d S reaction (baseline) to the 8d S reaction revealed differentially expressed genes in the 8d S reaction (Fig. 3F; Supplementary Table Ten). Induced genes included a beta-1,4-endoglucanase-3 precursor (AF044210.1), (FC: 9.3); a beta-1,4-endoglucanase-4 (AY043224.1), (FC: 9); a SCN esophageal gland cell protein (AF345801.1), (FC: 5.65); a ubiquitin extension protein (AF469060.1), (FC: 4.67) and a putative gland protein G33E05 (AF502392.1), (FC: 3.44). Suppressed genes were a C-type lectin domain protein (AF498244.1), (FC: -291); a hypothetical esophageal gland cell secretory protein 4 (AF273731.2), (FC: -236); the Y52B11A.8 gene (CB281657), (FC: -224); the putative cuticular collagen (CB378944), (FC: -127) and the hypothetical protein CBG11380 (CK394435), (FC: -110).

### Hierarchical clustering of gene expression across reaction types and time points

**Figure 4:**
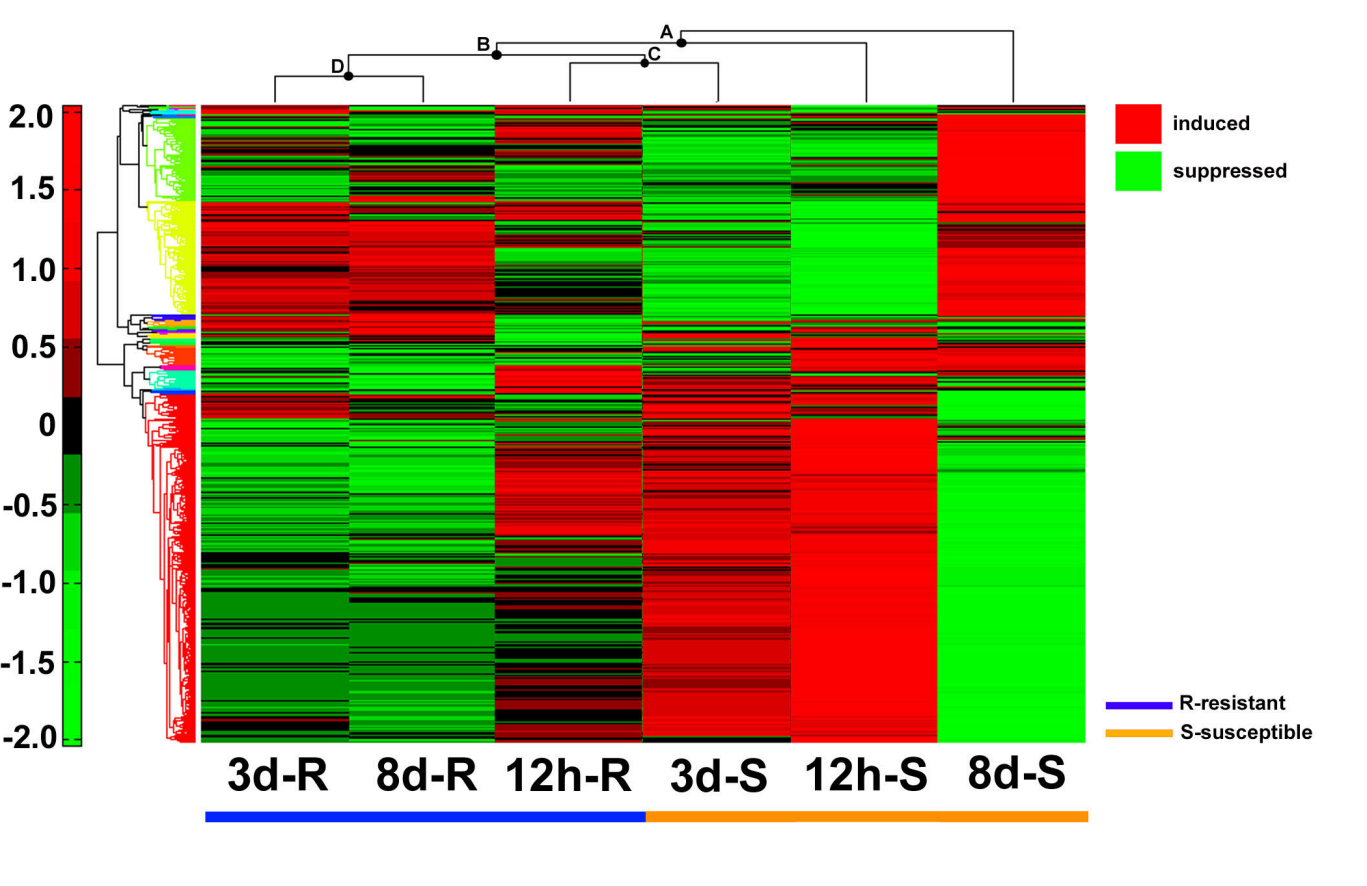
**Heat map of resistant and susceptible reactions**. Heat maps are arranged according to their hierarchical clustering as shown above the heat maps. Induced genes are represented in red. Suppressed genes are represented in green. Blue line (below heat maps) denotes resistant reactions. Orange lines (below heat maps) denote susceptible reactions. Samples, left to right are: 3d-R (3d-resistant [NL1-RHg]); 8d-R (8d-resistant [NL1-RHg]); 12 h-R (12 h-resistant [NL1-RHg]), 3d-S (3d susceptible [TN8]); 12 h-S (12 h- susceptible [TN8]); 8d-S (8d-susceptible [TN8]).

Hierarchical clustering using heat maps was used to develop a three time point (time series) analysis of *H. glycines *expression during both R and S reactions. The analysis compared R and S samples at T2, T3 and T4. The root of the heat map was the 8d S sample (Fig. 4, Supplementary Table Eleven). Node A was the branch point for the 12 h S sample (Fig. 4). Node B was the branch point for the subordinate nodes C and D. Nodes C and D each bifurcated. Node C was represented by the 3d S and 12h R reaction samples (Fig. 4). Node D was the branch point for the 3d and 8d R samples (Fig. 4).

### Gene expression as monitored during the course of R or S reactions

**Figure 5:**
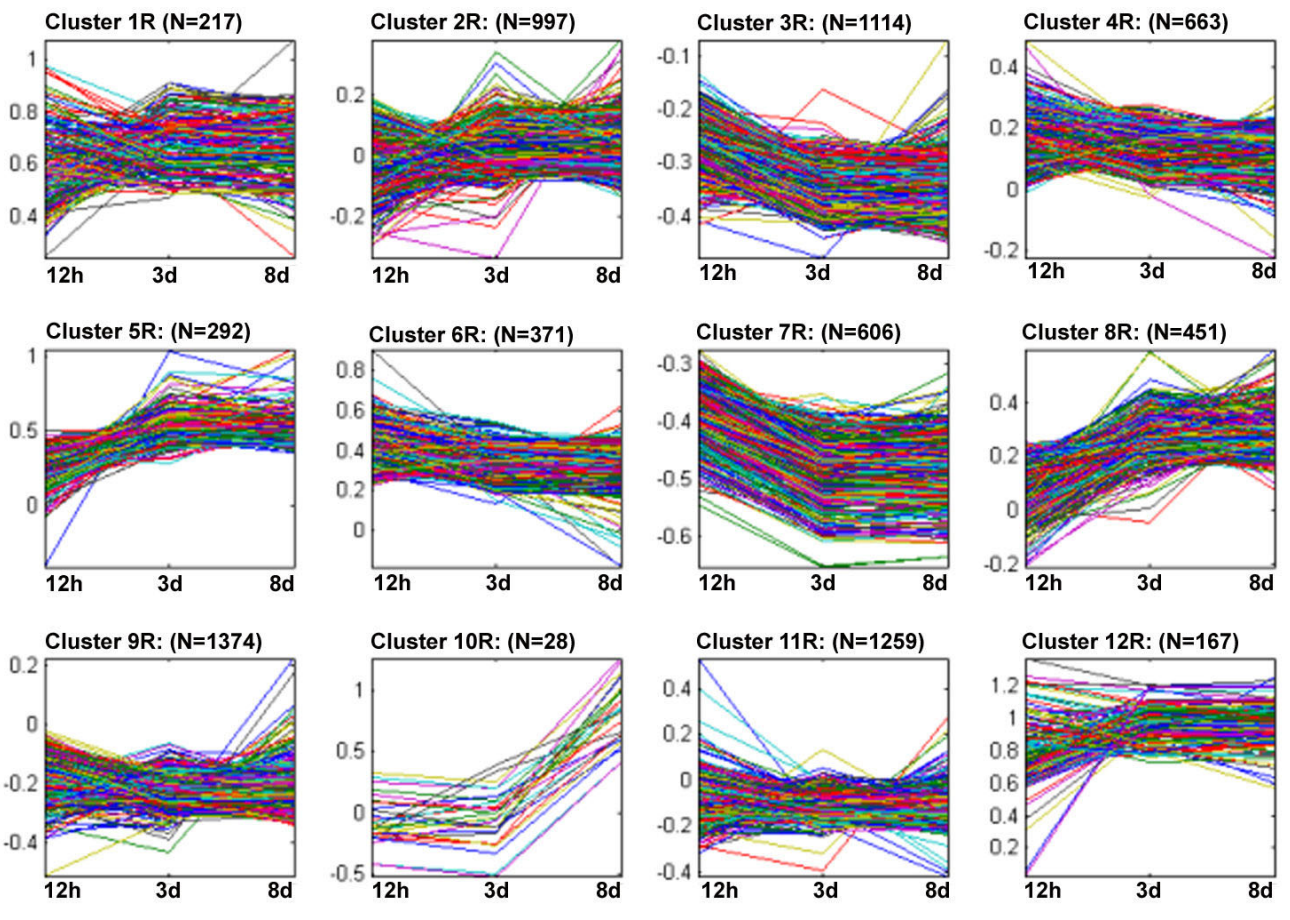
**K-means gene clustering for the resistant reaction based on expression profiles for 12 h, 3d and 8d time points**.

**Figure 6:**
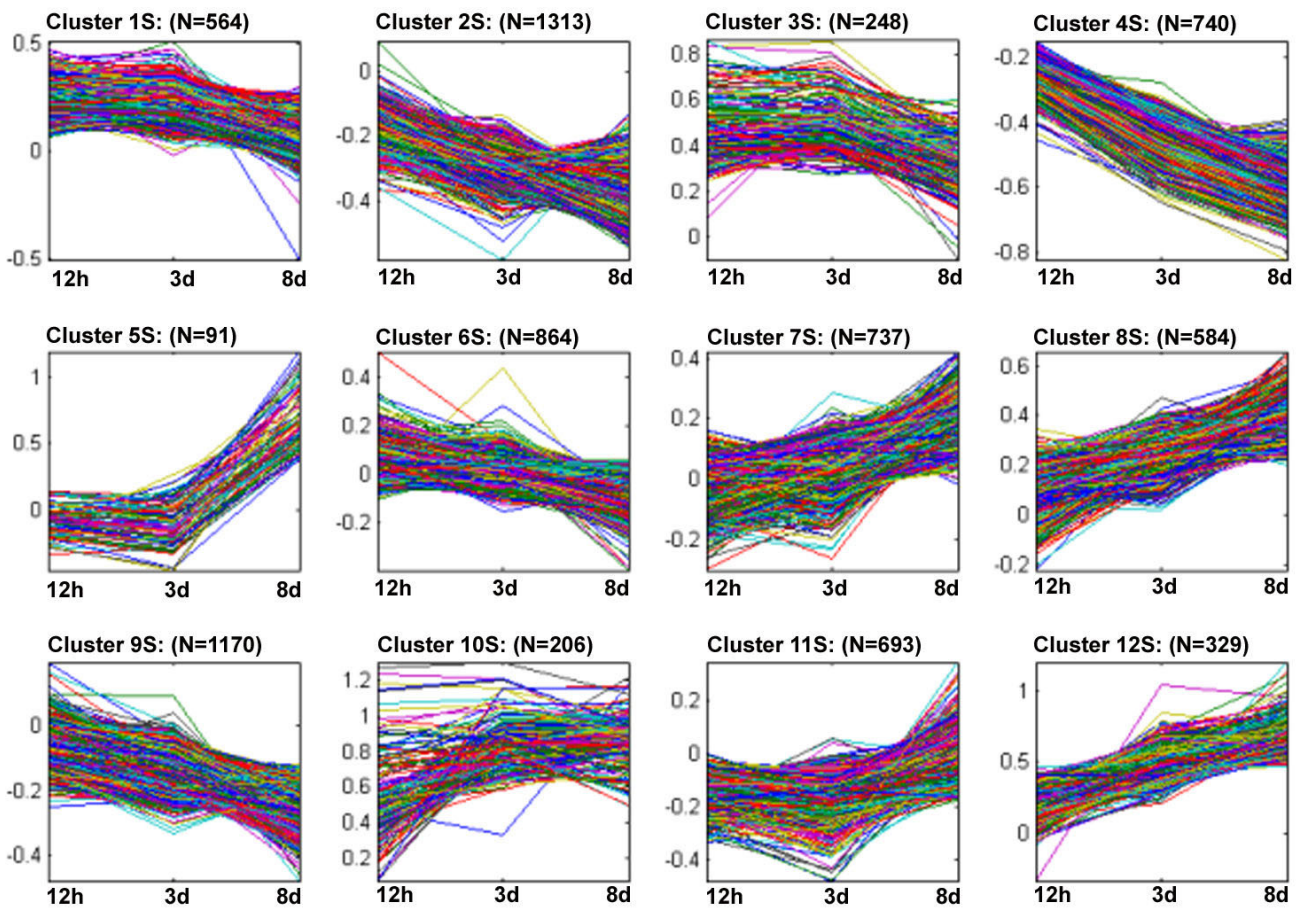
**K-means gene clustering for the susceptible reaction based on expression profiles for 12 h, 3d and 8d time points**.

K-means clustering was done on the log2 normalized expression data. It was used to group genes with similar expression profiles during the course of infection. This resulted in the identification of 12 clusters that were used to identify centroids to be used as a starting point for cluster identification. After 1000 repetitions, 12 gene clusters were identified so that all the values were affiliated with a specific cluster. Using more or fewer clusters resulted in either over-fitting or under-fitting the R (Fig. 5, Supplementary Tables Twelve to Twenty-three) or S (Fig. 6, Supplementary Tables Twenty-four to Thirty-five) datasets to a respective centroid.

Two R profile types stood out as meriting mention. Firstly, Figure 5-5R, is a profile consisting of genes that are initially expressed at lower levels at 12 h but then increase in their expression by 3 and 8d. Genes were sorted that had experienced their highest expression at the 8d time point. Two of these genes are the hypothetical protein Hgg-18 from *H. glycines *(CB935297) that has K-means expression values of 0.079 (12 h), 0.654 (3d) and 1.049 (8d). Another gene (P22U) was a homolog of the filarial heartworm nematode *Dirofilaria immitis *(CB825185) that has K-means expression values of 0.128 (12 h), 0.723 (3d) and 1.01 (8d). A second profile type, Figure 5-10R are genes that are lower in expression at the 12 h and 3d time points but then experience an increase in expression at 8d. Many of these genes are unknown. However, two of the top five genes do have homology to known genes. For example, one gene was a cuticular collagen (CB378944) originally identified from the plant parasitic nematode *Globodera pallida*. Its K-means expression values were 0.326 (12 h), 0.256 (3d), 1.154 (8d). A second gene was a C-type lectin domain protein identified from *H. glycines *(AF498244.1). Its K-means expression values were -0.168 (12 h), -0.149 (3d), 1.11 (8d).

### Gene expression: comparative time course analyses of gene expression during R or S reactions

**Figure 7:**
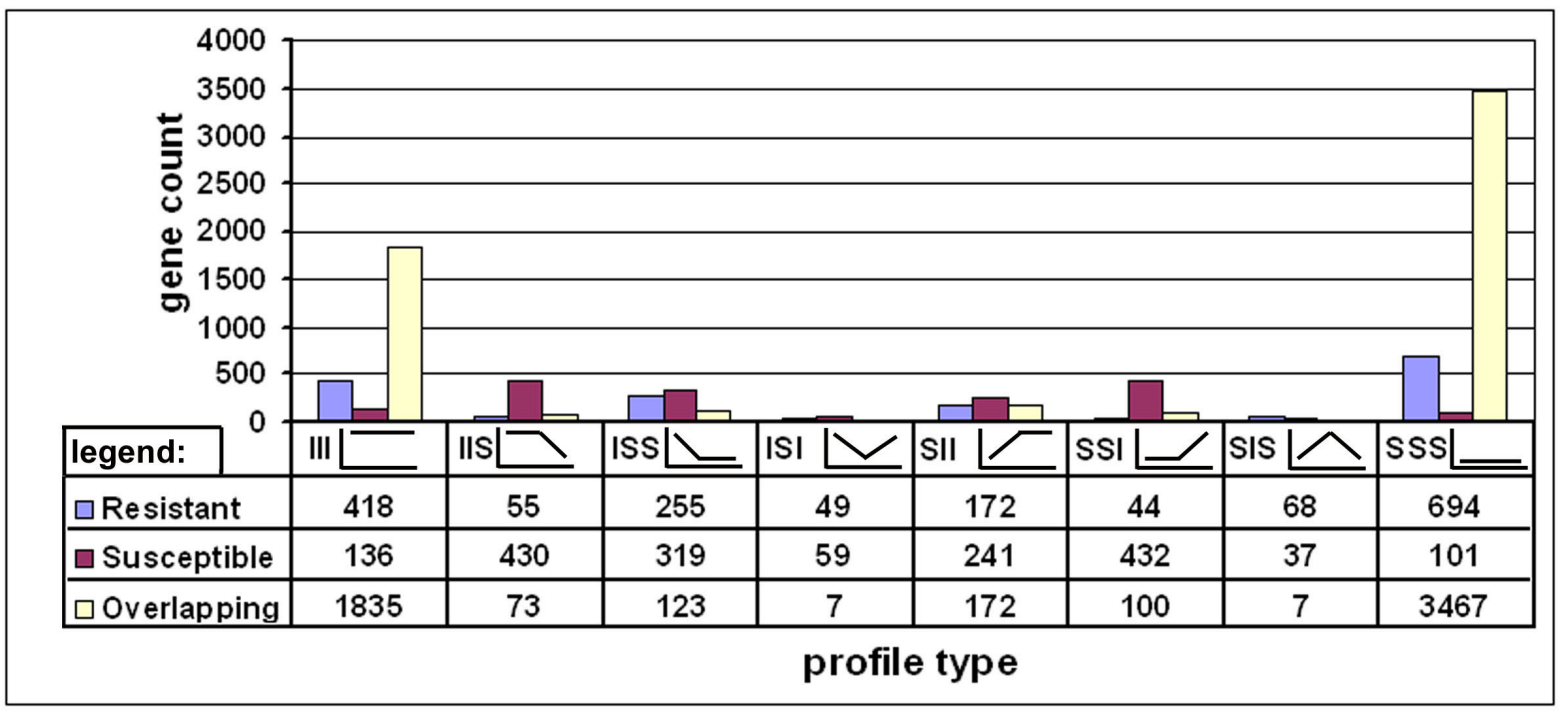
**Gene clustering based on expression profiles for 12 h, 3d and 8d time points having similar expression over the course of infection**. X-axis, profile histograms. Y axis, gene count. Blue, genes matching the profile and only found in the R reaction. Purple, genes matching the profile and only found in the S reaction. Yellow, genes matching the profile and found in both the R and S reaction (overlapping). The profile naming convention contains three letters, each for one of three successive time points, 12 h, 3d, 8d. I, induced; S, suppressed. Profile1: III, Profile 2: IIS, Profile 3: ISS; Profile 4: ISI; Profile 5: SII; Profile 6: SSI; Profile 7; Profile 8: SSS. The gene count is provided, graphically.

**Figure 8:**
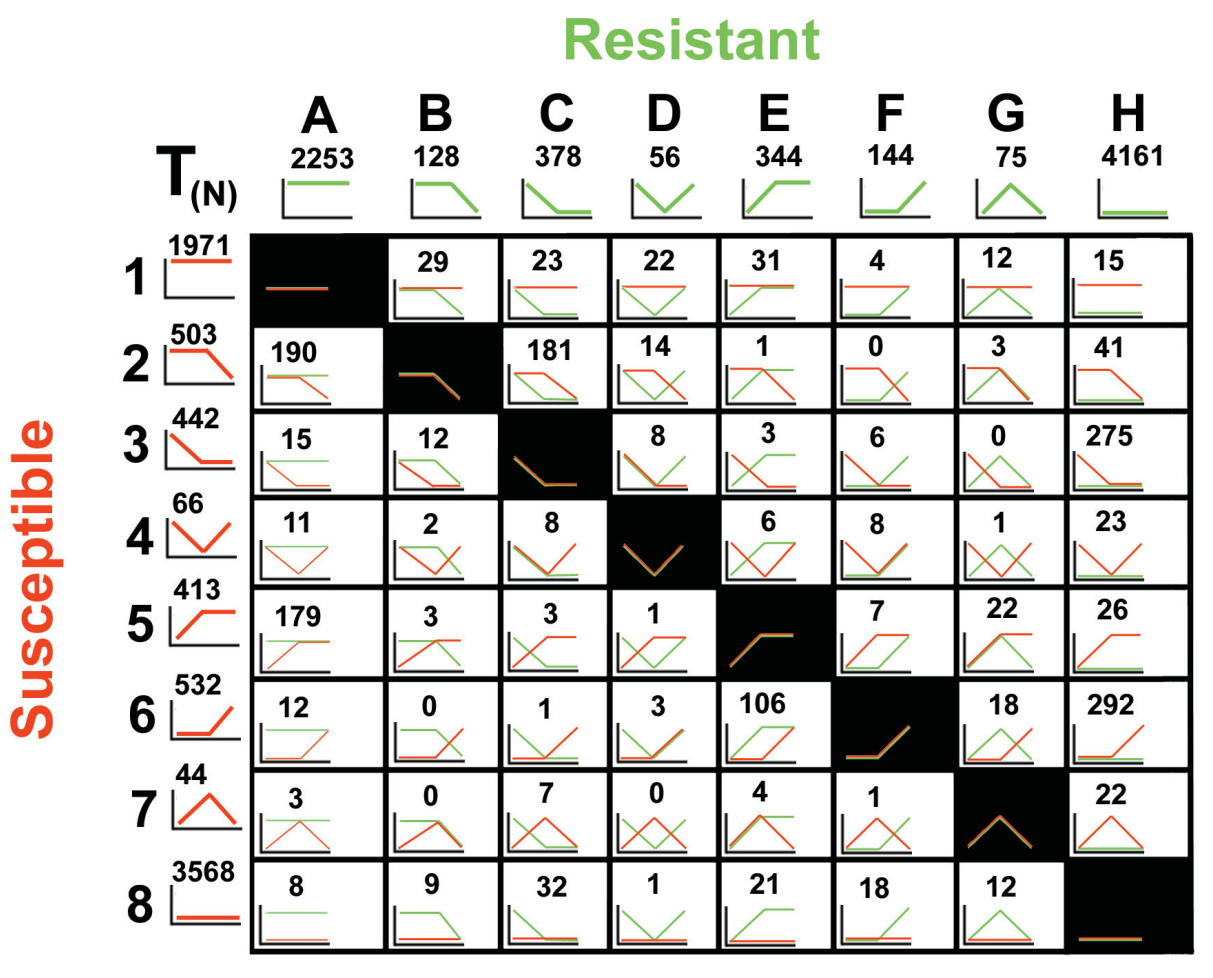
**Gene clustering based on expression profiles for 12 h, 3d and 8d time points having contrasting expression over the course of infection**. X-axis (green), Resistant profiles. Resistant: Profile A, III; Profile B, IIS; Profile C, ISS; Profile D, ISI; Profile E, SII; Profile F, SSI; Profile G, SIS; Profile H, SSS. Y-axis (red), Susceptible profiles. Susceptible Profile 1, III; Profile 2, IIS; Profile 3, ISS; Profile 4, ISI; Profile 5, SII; Profile 6, SSI; Profile 7, SIS; Profile 8, SSS. The gene counts for the contrasting gene expression analyses are provided. The gene counts for the overlapping profile pair were provided (Fig. 7). All gene lists are provided as supplemental data (Supplemental Table Thirty-seven).

Gene expression was monitored during the course of infection for *H. glycines *undergoing either R or S reactions. Genes undergoing similar expression profiles over time were identified for R and S reactions (Fig. 7, Supplemental Table Thirty-six). However, dissimilarity in gene numbers existed for R and S reactions (Fig. 7, Supplemental Table Thirty-six). This was further explored by identifying the numbers of genes that were overlapping in those similar profiles (Fig. 7, Supplemental Table Thirty-six). The remaining genes were unique to the R or S reaction type (Fig. 7, Supplemental Table Thirty-six). These observations demonstrated that a significant amount of genes were different between R and S reaction profiles. Therefore, R and S reaction profiles could be filtered per gene into contrasting gene expression (Fig. 8, Supplemental Table Thirty-seven). Several contrasting profiles (N = 6) had more than 100 genes in them. These profile types are A2, C3, H3, A5, E6 and H6 (Fig. 8, Supplemental Table Thirty-seven).

## Discussion

MA compared gene expression between populations of *H. glycines *nematodes experiencing either an R or S reaction. The analyses also compared different chronological time points during their life cycle. This analysis, however, was limited to the first 8 days of infection. This is because the R reaction, as evident by the presence of collapsing syncytia in Peking, is nearing its completion by this time under the experimental conditions used here [19,20].

### Selection of *H. glycines *populations in the analyses

The experiments presented here were aimed at obtaining *H. glycines *undergoing R or S reactions in the identical *G. max *genetic background. Importantly for the analyses, both NL1-RHg and TN8 were exposed to the same *G. max *genotypes during their maintenance in the lab and during the experiments. Prior to the experiments, NL1-RHg and TN8 were maintained on the *G. max *genotype Kent that undergoes an S reaction to both *H. glycines *populations. Thus, differences in gene expression observed in pi-L2 of NL1-RHg and TN8 are not caused by their maintenance on different plant genotypes or species.

*G. max *genotype Peking roots undergo an R reaction if infected with NL1-RHg and undergo an S reaction if infected with TN8. An important caveat is that while NL1-RHg does experience an R reaction in Peking, it is not a disarmed, non-pathogenic population of *H. glycines*. This is because, as mentioned before, NL1-RHg does experience an S reaction in Kent. Kent is a *G. max *genotype that does not engage in an R reaction. Therefore, NL1-RHg will experience an R or S reaction based off of the *G. max *genotype it infects. During the S reaction, NL1-RHg initiates the formation of a syncytium that it feeds from during the course of its life cycle in Kent. Prior experiments have shown that changes in Kent gene expression accompanies infection in whole infected roots [30] as well as syncytia isolated by laser capture microdissection (LCM) [38].

Conversely, NL1-RHg initiates the formation of a syncytium in *G. max *genotype Peking that then subsequently collapses during the R reaction. MA have revealed gene expression of whole, infected Peking roots undergoing an R reaction [28]. Subsequent analyses have compared R and S reactions [19]. The comparative analyses of R to S reactions allowed for the identification of Peking gene expression that was common or unique to each reaction [19]. A companion MA analysis has revealed gene expression of syncytia at 3d undergoing an R reaction [20]. This is a time point prior to their collapse during the R reaction.

### Gene expression in *H. glycines *populations undergoing R or S reactions is different before infection

The MA presented here were performed on pi-L2, comparing NL1-RHg to TN8, resulting in the identification of genes that are common and unique to each reaction. The analyses indicate that NL1-RHg and TN8 have measurable population-specific patterns of gene expression even before their infection of *G. max *genotype Peking. The investigation used pi-L2 NL1-RHg and TN8 that were grown and maintained identically on Kent. For the pi-L2 analyses, samples were obtained before the nematodes were placed on Peking. Thus, it is unlikely that the differences in gene expression that were observed resulted from the nematodes perceiving the presence of Peking and manifesting those differences as variations in transcriptional activity prior to infection. The draft genome of *Pristionchus pacificus*, a parasitic nematode of the oriental beetle *Exomala orientalis *in the United States and Japan have revealed differences between the four strains commonly used in evolutionary studies [47]. A whole genome comparison between the Washington and California strains resulted in the identification of a difference of 4.3% of all ungapped positions [47]. Thus, a substantial amount of genetic variation that would influence gene expression has been shown to exist in different strains (i.e., populations) of other parasitic nematodes.

The annotation of the probe sets here show that many of the genes that are induced in NL1-RHg and suppressed in TN8 possess homology to putative pharyngeal gland proteins [35,36]. The genus *Heterodera *has three pharyngeal glands. Two of the glands are subventral and one is dorsal. Many of the genes identified in previous experiments are subventral gland specific [48]. The subventral glands are very active. However, both their size and activity decrease dramatically during root penetration and their migration [48]. Importantly, this occurs before feeding cell initiation. Many of these genes, however, have been shown to have roles in weakening the cell wall as the nematodes migration through the root.

The large number of genes identified in this analysis demonstrates that population-specific expression patterns in gene activity are present even before the nematodes infect Peking. Thus, it appears as though the different nematode populations are equipped differently. In the experiments presented here, *H. glycines *NL1-RHg and TN8 had the opportunity to infect the same a *G. max *genotype (i.e., Peking), resulting in vastly different outcomes. Why *H. glycines *are equipped differently to contend with the root environment is not understood at the molecular level. However, these differences in gene expression may be so the various *H. glycines *populations can contend with the different genotypes of *G. max*. The results obtained in these analyses indicated that the array of parasitism genes involved in activities such as cell wall degradation may be much larger than previously appreciated. This was demonstrated by the recent genome sequencing project of the related plant parasitic nematode, *M. incognita *[3]. The analysis identified numerous secreted enzymes that target cellulose, xylan, arabinan or pectin that were previously unknown [3]. In addition to these, numerous expansin-like proteins were identified and in total, 81 genes were identified to be involved in these cell wall degrading processes [3].

An observation made in the analysis was the identification of several bacterial-like genes that are suppressed in pi-L2 NL1-RHg. Of note was a cell wall hydrolase (p-value of 0.0294, FDR of 11.61042) that most closely matched the gram-negative β-subgroup proteobacterium *B. multivorans *ATCC 17616. However, the probe set had just missed the cutoff set by the analysis method (i.e., fold change of ≥ |1.5|, p-value ≤ 0.05, FDR set at 10%). Regardless, the outcome was an important indicator of plant parasitic nematode biology. The recent sequencing project of *M. incognita *[3] revealed numerous horizontal gene transfers of plant cell wall degrading carbohydrate-active enzymes, expansins and invertases whose origins are likely to be bacterial [3]. The analysis presented here also identified a *H. glycines *β-expansin that was suppressed in NL1-RHg that experiences an R reaction. The identification of an induced fucosyltransferase family protein in NL1-RHg pi-L2 that experiences an R reaction was intriguing since multi-fucosylated cuticular structures are suggested to help nematodes avoid host detection [49]. These analyses demonstrate that gene expression is different in the two nematode populations before they had even contacted a potential host root. This outcome of the analysis is consistent with the other observations of differences in the genetic content of parasitic nematodes [25,47]). Of note, the putative parasitism gene chorismate mutase [25] was not observed to be differentially expressed between the two nematode races by our analysis methods.

A cDNA-amplification fragment length polymorphism (AFLP)-based strategy identified 22 out of 24,025 transcript-derived fragments (TDF) were differentially expressed in the root-knot nematode *M. incognita *[50]. The cDNAs were present in avirulent strains and absent in virulent near isogenic lines [50]. Of note, two were expressed specifically in the intestinal cells. One was expressed in the subventral esophageal glands. Two were expressed in the dorsal esophageal gland of L2. Such differences, as reflected by variations in gene expression, may be important as *H. glycines *evolve mechanisms to overcome naturally occurring resistance in different genotypes of *G. max*.

### Gene expression analyses at 12 h identify few changes in gene expression between nematode populations

MA demonstrated that the only differential gene expression occurring in NL1-RHg at 12 h is induced gene expression. Comparatively few genes were induced as well. Actin, a gene composing muscles is induced in nematodes experiencing an R reaction at 12 h. Prior analyses have shown that actin1 (*Hg-act-1*) is induced during the pi-L2 stage of development [46]. The analysis identified a *H. glycines *homolog of glutathione-S-transferase as being induces in *Hg-GST-1 *nematodes experiencing an R reaction at 12 h. GST is a component of the detoxification pathway. In *Meloidogyne incognita*, a secreted GST was shown to be induced 27 times more abundantly in the L3 stage than in the L2 [51]. However, in our case, an induced expression was noted in the i-L2 stage in *H. glycines *experiencing a resistant reaction as compared to those experiencing a susceptible reaction at 12 h. Another gene, carnitine palmitoyl transferase (CPT) family member (cpt-1) was identified as being induced in *H. glycines *experiencing a resistant reaction as compared to those experiencing a susceptible reaction at 12 h. The CPTase system is composed of two mitochondrial membrane-bound enzymes. The first is CPTase I and the second is CPTase II. The location of CPTase I is the inner side of the outer mitochondrial membrane. In contrast, the location of CPTase II is the inner mitochondrial membrane. These enzymes, along with acyl-CoA synthetase and the carnitine/acylcarnitine translocase, provide the mechanism for how long-chain fatty acids are transferred cytosolically to the mitochondrial matrix for β-oxidation. In humans, mutations in CPT are a clinically heterogeneous autosomal recessive disorder of energy metabolism. CPT I genetic defects impair liver activity while CPT II genetic defects are characterized, clinically, by muscle weakness and rhabdomyolysis (rapid breakdown of skeletal muscle) [52].

### Gene expression is similar in *H. glycines *populations experiencing an R or S reaction at 3d

Very little difference in gene expression was observed between NL1-RHg and TN8 at 3d. Histological analyses of root tissue development during R and S reactions at 3d have been performed. Those analyses have demonstrated that *H. glycines *initiates the formation of syncytia that appear similar at the anatomical level. While syncytia appear similar at the anatomical level at 3d, molecular differences are present [20]. Syncytia collected from Peking roots undergoing R or S reactions revealed differences in gene expression between the two reactions at 3d [20]. Some of those differences involved induced expression of genes involved in important aspects of the defense response. Some of the genes induced in syncytia undergoing an R reaction as compared to syncytia undergoing an S reaction were lipoxygenase, *G. max *HSP70 (GmHSP70), superoxide dismutase, WRKY transcription factor and GmRLK3 [20]. Therefore, localized changes in the feeding environment of the nematode (the syncytium) are underway even though they are not yet evident at 3d when examining NL1-RHg and TN8 gene expression.

### Suppression of gene activity occurs between 3 and 8d in *H. glycines *populations experiencing R

The analysis identified that a substantial difference in gene expression is present between the two nematode races as the development of R or S transitions from the 3d to the 8d time point. A smaller proportion of that difference in gene expression is induced genes. Most of the genes having positive BLASTX matches in Genbank were putative gland proteins. However, several other genes had *bona fide *biological roles. Some of those genes were ubiquitin extension protein [53] and a β-1,4-endoglucanase [36]. The role of these genes in infection is unclear since they are induced in NL1-RHg undergoing an R reaction in *G. max *roots. However, it is possible that the induced expression is due to the continued attempts at establishing a functional syncytium. Alternatively the genes are protective in nature.

A large proportion of the probe sets on the Affymetrix^® ^soybean GeneChip^® ^measured suppressed gene activity in NL1-RHg at 8d. Annotation of these genes identified steroid alpha reductase as the top hit having a positive BLASTX match. However, several more highly suppressed genes of unknown function were identified. Because parasitic nematodes do not synthesize sterols, they have a nutritional requirement that must be met by their host. Plant parasitic nematodes accomplish this by metabolizing phytosterols. The metabolism of these sterols includes saturation of the phytosterol nucleus and dealkylation of phytosterols at C24 [54]. Because of the nature of the analysis presented here, steroid alpha reductase is the most highly induced gene with a positive BLASTX match in TN8 experiencing an S reaction. This outcome is consistent with TN8 actively engaging in feeding at 8d.

Other genes were also suppressed in NL1-RHg. The second highest match was a serine proteinase (sp-III gene) [55]. Three serine proteinases have been isolated from *H. glycines *and their activity determined [55]. CpTI, a serine proteinase inhibitor, was expressed in transgenic potato (*Solanum tuberosum*) and shown to suppress the early growth of the potato cyst nematode *Globodera pallida *[56]. The identification of two highly suppressed genes in NL1-RHg experiencing an R reaction at 8d suggests that the feeding activity or machinery of these nematodes may be compromised. Alternatively, those genes never become induced in NL1-RHg since all of the developmental prerequisites prior to the induction of those genes involved in feeding have not been met. Regardless, this outcome is consistent with the observation that NL1-RHg are not increasing in girth between 3 and 8d.

### *H. glycines *gene expression comparing different time points within R or S reactions

Numerous differential expression analyses are presented demonstrating gene expression in *H. glycines *experiencing R or S reactions at single time points. Other analyses demonstrated gene expression in *H. glycines *experiencing R or S reactions during the course of infection. Many of these analyses demonstrated commonalities in expression for *H. glycines *experiencing either an R or S reaction. While it would be intriguing to speculate how they function during R, many of these genes are also found to be induced during each of the three S reaction comparative analyses (i.e., 12 h vs. 3d, 12 h vs. 8d and 3d vs. 8d). Thus, the induced gene expression presented in those analyses appears to be common to the two reaction types.

Genes experiencing a substantial suppression of activity within a reaction type (i.e., R or S) were several cuticular collagens. The cuticle would be expected to present molecules that can be perceived by plant cells during its defense response. The suppression of these genes may be an indicator that the plant is impairing cuticle maintenance at 3d during the R reaction. While it would be interesting to speculate how they function during the R reaction, many of these genes (i.e., cuticular collagens) are also found to be suppressed during each of the three comparative analyses for the S reaction (i.e., 12 h vs. 3d, 12 h vs. 8 h and 3d vs. 8d). Thus, the identification of induced gene expression in those analyses appears to be common to the two reaction types. This highlights why analyses addressing the differences in gene expression that is present between the *H. glycines *populations during infection are important. Such analyses, as presented here, may better explain the nature of the R or S reaction during infection of the same *G. max *genotype. These sorts of analyses may also be used much in the same way as mutant analyses are used to identify genes involved in a specific process (i.e., R or S). Therefore, deficiencies in gene expression in a particular nematode population would then be revealed and correlated to a particular outcome (i.e., R or S). The recent sequencing of a portion of the *H. glycines *genome [25], thus can be used as a guide to better understand the nature of expression differences as observed in our analyses.

### Contrasting gene expression as *H. glycines *experiences R or S

A time course analysis of contrasting *H. glycines *gene expression was presented. The comparison demonstrated gene expression as *H. glycines *experienced an R or S reaction. Six of these profile types had greater than 100 genes each. Comparing their expression with *G. max *gene expression during an R reaction may identify deficiencies in those *H. glycines *genes involved in defense. It may also explain how the nematodes are being overcome as the *G. max *defense response is becoming established. For example, in whole roots undergoing R, a substantial number of *G. max *probe sets for HSPs (i.e., ClpB/HSP101, HSP90 and HSP70), LOX and genes involved in energy metabolism measured induced gene expression [19]. Laser capture microdissection and MA analyses of syncytia undergoing a resistant reaction resulted in the identification of genes known to be involved in various defense responses (i.e., HSP70, WRKY-like transcription factor, GmRLK3, Superoxide dismutase and LOX) [20]. Therefore, *G. max *gene expression at the site of infection and distally in the cortex tissue surrounding *H. glycines *is likely playing roles during the R reaction.

### Functional analyses

The resultant analysis identified signature gene expression that is occurring in the NL1-RHg and TN8 populations during the R or S reaction, respectively. Of note throughout these analyses was the observation that a large proportion of genes do not have significant matches in the public databases. This underscores the necessity for functional analyses of these genes. One promising method for functional analyses in *H. glycines *has been the use of RNA interference (RNAi) [57]. Functional analyses of *H. glycines *genes using RNAi has been used to demonstrate essential roles for some genes [58]. Other analyses have investigated genes that are putatively involved in parasitism. However, of the many dorsal esophageal or subventral gland putative parasitism genes targeted in these RNAi screens, few if any have identified any essential role [59-61]). Those results may point to their combinatorial or additive role during infection. The outcomes of those experiments would be in agreement with the observations presented here. For example different suites of putative parasitism genes appear to be expressed in the NL1-RHg and TN8 populations even after they have each engaged in an S reaction during their maintenance in the *G. max *genotype Kent. Our experiments have identified many genes that are even more highly expressed than the putative parasitism genes that have no identifiable homolog in the public databases. This may be a suite of genes to explore further. Alternatively, the essential parasitism genes have not yet been isolated and functionally tested in ways that can truly reveal their function. For example, the recent sequencing of the related plant parasitic nematode *M. incognita *has revealed that while many components of the RNAi pathway are present (i.e., *ego-1*, *rrf-1*, *rrf-2 *and *rrf-3*), those that are essential for the systemic spreading mechanism (i.e., *sid-1*, *sid-2*, *rsd-2 *and *rsd-6*) do not have identifiable homologs [3]. Similar observations have been made in the analysis of the sequenced genome of the filarial nematode parasite *Brugia malayi *[62]. This may explain some of the ambiguity in the findings of plant parasitic nematode RNAi screens [60,61]. Analyses that have produced large amounts of DNA sequence information in *H. glycines *have not yet determined whether these homologs exist [25]. Thus, further annotation of those DNA sequences may reveal whether identifiable homologs exist in *H. glycines*.

## Conclusion

The work presented here identified gene expression that occurs as *H. glycines *pi-L2 nematodes infect *G. max *roots, resulting in R or S reactions. The work also provides gene expression data of *H. glycines *during infection of a *G. max *genotype (i.e., Peking) that possesses resistance genes. The work provides information on *H. glycines *gene expression that occurs as *G. max *resistance is overcome. Thus, the work may help identify how populations of *H. glycines *that can lie dormant for years in the field as eggs within cysts successfully evolve mechanisms to overcome resistance. This work may also provide insight for strategies aimed at engineering resistance to *H. glycines*. The functional analysis of some of these genes has been performed with the aim of engineering *G. max *resistance to *H. glycines *[63]. This is particularly important for controlling *H. glycines *populations that have developed ways to overcome the limited resistance afforded by various genotypes of *G. max *such as Peking. These results demonstrate that caution should be employed when using any single nematode species or population as a model for parasitic nematode research. The diversity of gene expression that was obtained in these analyses warrants further exploration. The results demonstrate that it is also important to study homologous systems when the goal is engineering nematode resistance into a specific plant species.

## Authors' contributions

VPK, designed the study, analyzed data, wrote manuscript, revised manuscript; PH, performed microarray analyses, revised manuscript; MHM, designed the study, managed plants and nematodes, isolated samples, revised manuscript; NWA, performed statistical analyses of microarray analyses, revised website and manuscript; BFM, designed the study, contributed to writing and editing of the manuscript.
